# Biotemplated Synthesis of Anatase Titanium Dioxide Nanoparticles via Lignocellulosic Waste Material

**DOI:** 10.1155/2014/205636

**Published:** 2014-07-15

**Authors:** Donya Ramimoghadam, Samira Bagheri, Sharifah Bee Abd Hamid

**Affiliations:** Nanotechnology & Catalysis Research Centre (NANOCAT), University of Malaya, IPS Building, 50603 Kuala Lumpur, Malaysia

## Abstract

Anatase titanium dioxide nanoparticles (TiO_2_-NPs) were synthesized by sol-gel method using rice straw as a soft biotemplate. Rice straw, as a lignocellulosic waste material, is a biomass feedstock which is globally produced in high rate and could be utilized in an innovative approach to manufacture a value-added product. Rice straw as a reliable biotemplate has been used in the sol-gel method to synthesize ultrasmall sizes of TiO_2_-NPs with high potential application in photocatalysis. The physicochemical properties of titanium dioxide nanoparticles were investigated by a number of techniques such as X-ray diffraction analysis (XRD), transmission electron microscopy (TEM), Fourier transform infrared spectroscopy (FTIR), Raman spectroscopy, thermogravimetric analysis (TGA), ultraviolet visible spectra (UV-Vis), and surface area and pore size analysis. All results consensually confirmed that particle sizes of synthesized titanium dioxide were template-dependent, representing decrease in the nanoparticles sizes with increase of biotemplate concentration. Titanium dioxide nanoparticles as small as 13.0 ± 3.3 nm were obtained under our experimental conditions. Additionally, surface area and porosity of synthesized TiO_2_-NPs have been enhanced by increasing rice straw amount which results in surface modification of nanoparticles and potential application in photocatalysis.

## 1. Introduction

Titanium dioxide is an important n-type wide band-gap semiconductor with light absorbing, charge transport, and surface adsorption properties [1]. Three different crystallite structures of brookite, anatase, and rutile have been found for titanium dioxide [2]. Due to the exclusive properties like photoactivity, photostability, being chemically and biologically inert [3], being relatively inexpensive, and high stability [4], titanium dioxide has wide variety of applications including photocatalysis, catalysis, dye sensitized solar cells, and photovoltaic and water-splitting devices [5]. Therefore, exponential growth in research focuses on synthesis, properties, and applications of TiO_2_ nanostructures that have been accomplished in recent years.

There are various methods to synthesize titanium dioxide nanostructures such as chemical vapor deposition, microemulsion, chemical precipitation, hydrothermal crystallization, and sol-gel [3, 5]. Sol-gel is one of the most successful techniques to fabricate high photocatalytic titanium dioxide nanostructures [6], with controlled shape and porosity. Moreover, other advantages such as versatile process [7] with high purity, good homogeneity, and low processing temperature [8] can be taken into account for this synthetic technique.

Recently, synthetic methods of TiO_2_ nanostructures were accompanied with template-assisted approaches. The templating method is one of the frequently used methods to modify the surface properties of nanomaterials [9]. The surface modification of nanomaterials is mostly applied due to avoiding the agglomeration, increasing the stability and compatibility in different media. It also can assist in creating active sites on the nanomaterials' surface and eventually enhancing their activity. Generally templates are categorized into two major groups of soft and hard templates. Porous solids can be considered as hard templates such as anodic aluminum oxide (AAO) membranes, colloid beads, ordered mesoporous inorganic materials, and zeolites. Conversely, soft templates consist of mostly organic molecules, long-chain polymers, supermolecular aggregates, structure-directing agents, surfactants, gels, micelles, and different types of biological species. Soft templates in order to have special anisotropic structure not only render more sufficient synthetic process but also have the ability to be easily removed. In addition, it is proven that the effective and high quality encapsulation technique is feasible by soft templating. A variety of materials can be exploited as templates such as nanoporous materials, molecules and supramolecules, colloids, particles, and even biological species named as biotemplates [10].

Waste valorization is a term used for managing waste in the most sustainable way which has attracted a significant amount of attention in recent years [11]. Vast choices of advanced technologies are available to employ the agricultural wastes into novel functional nanomaterials [12]. The rice cultivation procedure results in two types of residues, straw and husk, of which a variety of valuable chemicals can be derived. Rice straw is the stalk of the rice plant that is left over as waste products on the field upon harvesting of the rice grain (i.e., the seeds of rice straw). Rice husk, the main byproduct from rice milling, accounts for roughly 22% of paddy weight, while rice straw to paddy ratio ranges from 1.0 to 4.3. Although the technology for rice husk utilization is well-established worldwide, rice straw is sparingly used as a source of renewable energy [12, 13]. In this regard, recently a lot of efforts have been carried out for conversion of various edible or nonedible biomass feedstocks into biofuels [14].

Various templates have been reported to synthesize metal oxide nanostructures. Moreover, surfactants as subsidiaries of templates have been widely used in the preparation of different nanoparticles with good size distribution and dispersity [15]. Adding diverse surfactants as capping agent into the reaction matrix can help synthesize monodispersed TiO_2_ nanoparticles [16]. TiO_2_ nanorods can be synthesized with different sizes and shapes through aid of surfactants. Different amines were applied to synthesize TiO_2_ nanomaterials as shape controller [16–19]. Another study has reported using sodium stearate and sodium oleate which could change the TiO_2_ nanoparticles from round-corner cubes to sharp-edged cubes [20]. In addition, the size distribution of TiO_2_ nanorods was largely controlled by the size distribution of anodic alumina membrane (AAM) [19–21]. Moreover, exploiting surfactants such as ammonium carboxylate perfluoropolyether and poly(dimethylaminoethyl methacrylate-block-1H,1H,2H,2H-perfluorooctyl methacrylate) led to increasing the crystal size [22].

Biological materials such as biomolecular structures, viruses, proteins, and DNA have attracted a lot of attention recently. Many studies have investigated the effects of different biotemplates on the synthesis and properties of different metal oxide nanostructures. Gelatin [23, 24], gum [25], starch [22–24, 26], rice straw [27], eggshell membrane [28], bamboo membrane [29], DNA [30], yeast, dandelion pollen, and albumen [28, 29] were all investigated to synthesize metal oxide nanoparticles such as ZnO, TiO_2_, and Ag nanoparticles. Applying rice straw as a soft biotemplate appears to be a promising way to synthesize titanium dioxide nanoparticles. Moreover, to the best of our knowledge, no such study on the synthesis of TiO_2_-NPs using rice straw as biotemplate is found in the open literature. Therefore, in this study, we investigate the effects of rice straw powder on properties of synthesized TiO_2_ nanoparticles via soft, inexpensive, and green template.

## 2. Experimental

### 2.1. Materials

All chemicals used in this work were of analytical reagent grade and used as received without any further purification. All the aqueous solutions were prepared using deionized water. Titanium (IV) isopropoxide 98% which was used as a main precursor was purchased from Acros Organics Co., and acetic acid 100% was purchased from Merck Co., Germany. Rice straw was purchased from local market and ground into powder form in milling machine, Fritsch Pulverisette 6 type planetary monomill, Germany.

### 2.2. Characterization

Powder X-ray diffraction (PXRD) analysis was performed on a Shimadzu diffractometer, XRD-6000 (Tokyo, Japan) equipped with CuK*α* radiation. The morphology of the titanium dioxide particles was characterized by transmission electron microscopy (TEM) LEO LIBRA-120, Carl Zeiss AG Company (Oberkochen, Germany). Particle size distribution has been calculated using Image J and SPSS software. Surface characterization of the material was carried out using nitrogen gas adsorption-desorption technique at 77 K using Autosorb-6B Quantochrome (FL, USA). Thermogravimetric and differential thermogravimetric analysis (TGA-DTG) were carried out using a Mettler Toledo instrument (Greifensee, Switzerland) using a heating rate of 10°C/min, in the range of 25–1000°C under nitrogen atmosphere. Fourier transform infrared spectra were recorded over the 400–4000 cm^−1^ range using a Perkin-Elmer 100 spectrophotometer (Waltham, MA, USA) under standard conditions. The structural properties were also investigated by inVia Raman microscope, Renishaw (Gloucestershire, UK), in the range of 100–700 cm^−1^. The UV-VIS-NIR spectrophotometer UV-3600 SHIMADZU was used to determine the optical properties.

### 2.3. Synthesis of TiO_2_ Nanoparticles

In a typical procedure [31], titanium (IV) isopropoxide was dissolved in deionized water (18.2 MΩ cm) and acetic acid with the molar ratio of 1 : 200 : 10. Glacial acetic acid acts as a chelating agent to prevent titanium isopropoxide from the nucleophilic attacks by the water. The solution was stirred for few hours and then different concentrations of rice straw powder 0, 0.25, 0.5, 1, 2, and 4 g were introduced into the solution (the ratio of titanium (IV) isopropoxide to rice straw powder was chosen at 1 : 0.1, 1 : 0.2, 1 : 0.4, 1 : 0.8, and 1 : 1.5 w/w%) and stirred. The mentioned solution was heated at 80°C until formation of the gel. The obtained gel was dried in the oven at 80°C overnight. Finally the dried gel was ground and calcined in a muffle furnace at 500°C for 5 hours.

## 3. Results and Discussion

### 3.1. Powder X-Ray Diffraction

**Figure 1:**
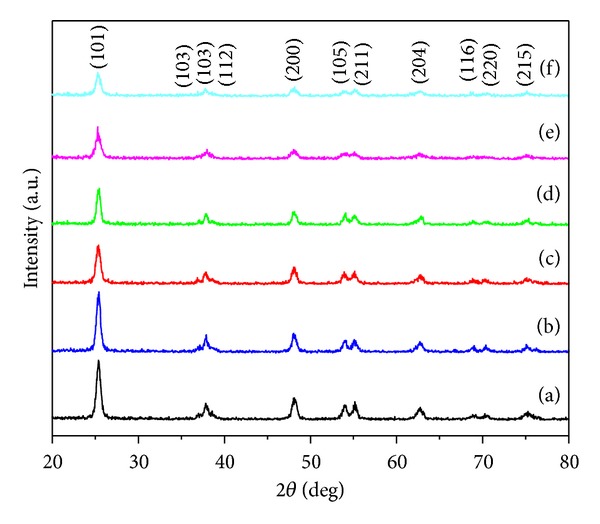
XRD patterns of TiO_2_-NPs using different concentrations of rice straw; (a) 0R, (b) 0.25R, (c) 0.5R, (d) 1R, (e) 2R, and (f) 4R (0R, 0.25R, 0.5R, 1R, 2R, and 4R refer to synthesized TiO_2_-NPs using 0.0, 0.25, 0.5, 1, 2, and 4 g rice straw, resp.).

To determine the phase of the produced TiO_2_-NPs, samples were examined by X-ray powder diffraction (XRD).  Figure 1 shows the XRD patterns of the TiO_2_ samples synthesized using different concentrations of rice straw powder after calcinations at 500°C for 5 hours. As clearly seen from Figure 1, all the diffraction peaks of the pure TiO_2_ prepared by the conventional sol-gel method can be indexed as anatase (Anatase XRD JCPDS Card number 78-2486). However, Budi et al. [32] reported mesoporous synthesis of titania using starch through sol-gel method involving mixed phases of anatase and rutile applying different concentrations of potato starch.

According to Figure 1, the diffraction peaks of as-synthesized TiO_2_ samples were broadened by increasing the concentration of rice straw. This is due to the decrease in the crystalline size. Moreover, no characteristic peaks can be observed in the XRD pattern for rice straw components. The crystalline sizes of the TiO_2_-NPs were determined by means of an X-ray line-broadening method by Scherer's formula (*D* = *Kλ*/*β*cos⁡⁡*θ*) where *λ* is the wavelength of X-ray radiation (CuK*α* = 0.15406 nm), *K* is a constant taken as 0.9, *β* is the line width at half maximum height (FWHM) of the peak, and *θ* is the diffracting angle. The (101) plane with highest intensity peak was chosen to calculate the crystalline size. The average crystalline size of synthesized TiO_2_-NPs with different concentrations of rice straw and also without rice straw is listed in Table 1. It is noteworthy to mention that 0R, 0.25R, 0.5R, 1R, 2R, and 4R refer to synthesized TiO_2_-NPs using 0.0, 0.25, 0.5, 1, 2, and 4 g rice straw, respectively.

### 3.2. Transmission Electron Microscopy

**Figure 2:**
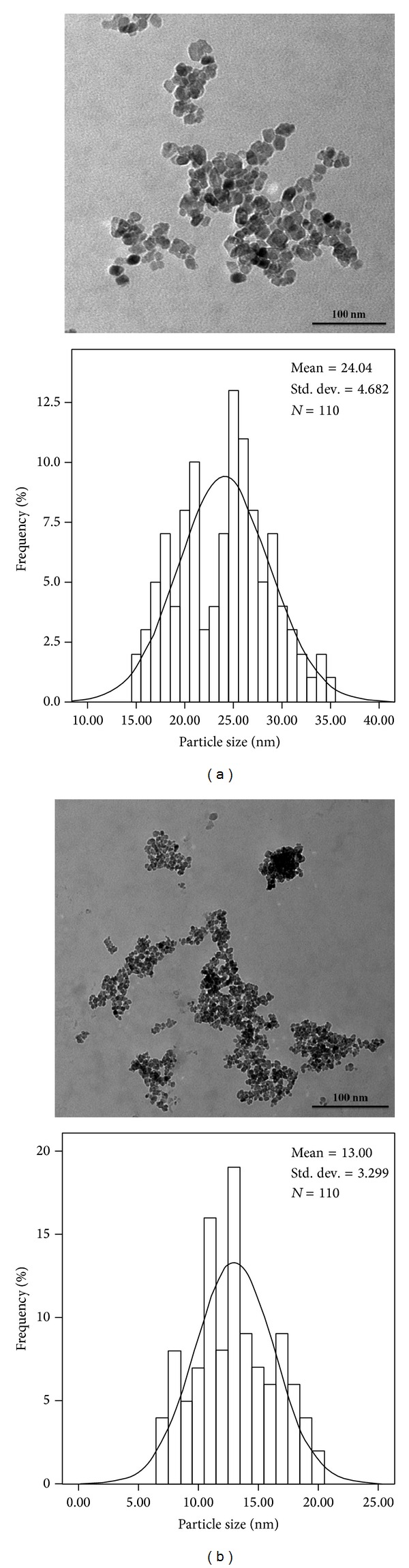
TEM images of TiO_2_-NPs prepared using (a) 0R and (b) 4R.

**Figure 3:**
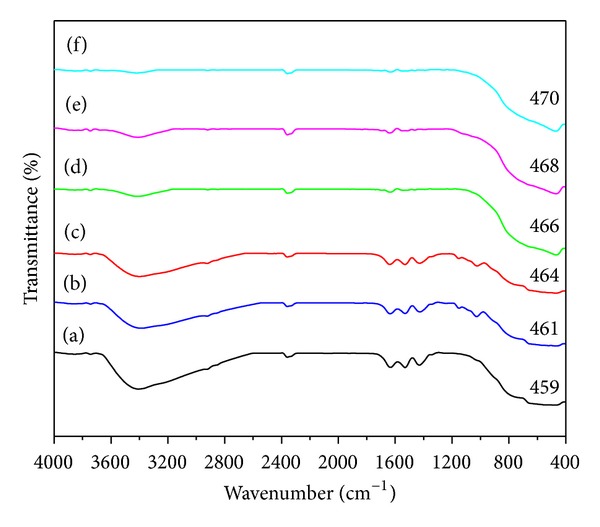
FTIR spectra of TiO_2_-NPs synthesized using (a) 0R, (b) 0.25R, (c) 0.5R, (d) 1R, (e) 2R, and (f) 4R.

In order to understand the effects of biotemplate on the size and morphology of the synthesized TiO_2_-NPs, TEM examination was conducted.  Figure 2 shows the TEM images and size distributions of the TiO_2_-NPs with and without rice straw after calcination at 500°C for 5 hours. The size histograms of the TiO_2_-NPs are shown below the relative TEM images. As seen from histograms in Figure 3, the mean particle size of TiO_2_ sample prepared without and with 4 g rice straw as biotemplate is 24.0 ± 4.7 nm and 13.0 ± 3.3 nm, respectively. It is notable that size of TiO_2_-NPs synthesized using rice straw considerably decreased compared to TiO_2_-NPs synthesized without rice straw (all results not shown here). The obtained result from TEM is in agreement with XRD results, representing that smaller size of TiO_2_-NPs can be obtained using rice straw as biotemplate which is acting like a directing agent.

Cellulose is the main component of rice straw consisting of mostly polysaccharides. Polysaccharides interfere in various stages of the titanium dioxide synthesis. The biopolymer, which is dispersed in the liquid media, behaves like an organic matrix, binding through their functional groups (hydroxylic or carboxylic groups) to many titanium ions. The initial association of the titanium ions to polysaccharide determines a homogenous dispersion of the ion in well confined spaces. After the change of the initial reaction conditions, in the presence of a precipitation agent, these binding positions provide preferred nucleation and growth sites for the hydrolyzed Ti^4+^-containing particles, due to the high local suprasaturation in titanium ions. The conversion of oxide precursor to oxide needs heating treatments. The homogeneous dispersion of the cations within the polysaccharide matrix and the low temperature of its thermal degradation shift the balance between nucleation and growth toward the formation of a larger number of smaller oxide crystals [33].

### 3.3. Fourier Transform Infrared and Raman Spectroscopy

Fourier transform infrared spectroscopy was used to investigate the effects of rice straw as biotemplate on the chemical properties of the TiO_2_-NPs prepared by sol-gel method. FTIR spectra were obtained at room temperature in the range of 4000–400 cm^−1^. Figure 3 shows the FTIR spectra of TiO_2_-NPs synthesized using different concentrations of rice straw as a biotemplate. In addition, spectra of TiO_2_-NPs sample synthesized using no rice straw are also represented for better comparison.

The bands centered at 1635 cm^−1^ and 3400 cm^−1^ are attributed to *δ*-H_2_O bending and vibration of hydroxyl groups [34]. Band at about 2357 cm^−1^ is assigned to Si–H stretching vibration caused by rice straw components. It is noteworthy to mention that the silicon compound may coexist with other compositions in the rice straw due to the presence of rice straw husk. In addition, absorption bands at around 1430 and 1530 cm^−1^ are attributed to the C–H bending and angular deformation of C–H bond in starch molecule, respectively [35]. The band at about 460 cm^−1^ is assigned to O–Ti–O in anatase phase which can be clearly observed in all synthesized TiO_2_-NPs samples. A closer look on the FTIR spectra of TiO_2_ samples synthesized using different concentrations of rice straw indicates a slight shift to higher wavenumbers in characteristic band of TiO_2_ compared to the sample synthesized using no rice straw. This shifting can occur due to the decrease in the particle size of TiO_2_-NPs.

**Figure 4:**
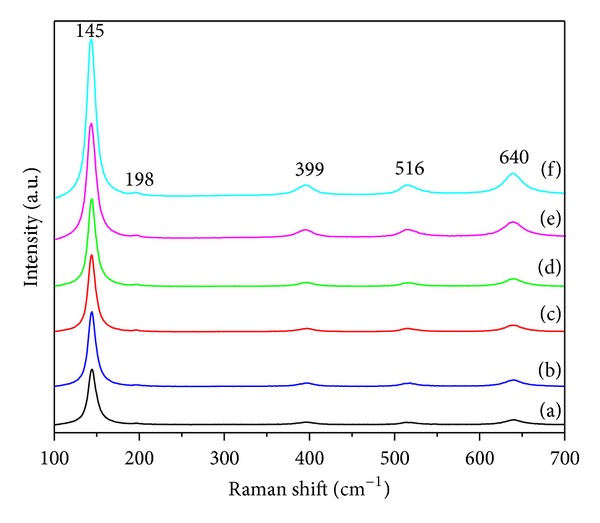
Raman spectra of TiO_2_-NPs synthesized using (a) 0R, (b) 0.25R, (c) 0.5R, (d) 1R, (e) 2R, and (f) 4R.

The structural properties of the TiO_2_-NPs were further investigated by Raman spectroscopy.  Figure 4 shows the Raman spectra of TiO_2_-NPs synthesized using different concentrations of rice straw as biotemplate along with spectra of TiO_2_-NPs sample synthesized using no rice straw as reference. All samples exhibit five distinct Raman-active modes of *E*_*g*_ (145 cm^−1^), *E*_*g*_ (198 cm^−1^), *B*_1*g*_ (399 cm^−1^), *A*_1*g*_ (516 cm^−1^), and *E*_*g*_ (640 cm^−1^) for anatase TiO_2_ [36] calcined at 500°C, verifying the materials' phase composition determined by XRD and TEM. Analysis of the Raman spectra by multipeak fitting revealed appreciable shifts of the anatase Raman bands for synthesized TiO_2_-NPs using different concentrations of rice straw compared to the reference sample. This behavior is characteristic of size effects raised by biotemplate [31]. Specifically, the lowest frequency and most intense *E*_*g*_ anatase mode shifted from 144 cm^−1^ for the reference to 145 cm^−1^ which is qualitatively consistent with the decrease of the anatase crystalline size.

### 3.4. Thermogravimetric Analysis

**Figure 5:**
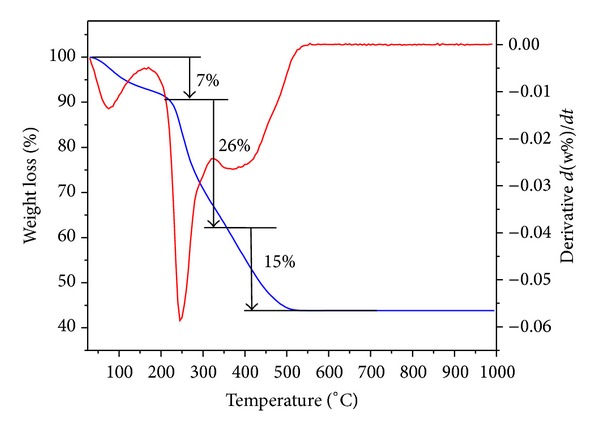
TGA-DTG thermogram of as-synthesized TiO_2_-NPs samples synthesized using 4 g rice straw (4R).

The thermogravimetric and derivative thermogravimetric analysis (TGA/DTG) have been investigated on the TiO_2_-NPs synthesized by the sol-gel method using different concentrations of rice straw.  Figure 5 shows the TGA-DTG curve of TiO_2_-NPs synthesized using 4 g rice straw. It can be clearly seen that TG curve descends until it becomes horizontal around 500°C. The TG and DTA traces show three main regions. The first weight loss below 100°C (7%) is assigned to dehydration of water. The second weight loss from 200 to 300°C (26%) is attributed to the decomposition of rice straw components which are mainly carbohydrates. Similar results were also reported by Ramimoghadam et al. [27]. It is noteworthy to mention that the weight loss percentages of 4, 5, 8, and 15% were observed for TiO_2_-NPs synthesized using 0.25, 0.5, 1, and 2 g rice straw. The third step from 350 to 500°C (15%) is related to both the decomposition of the organic group's residuals and the condensation of the TiO_2_ anatase phases.

### 3.5. UV-Visible Spectroscopy

**Figure 6:**
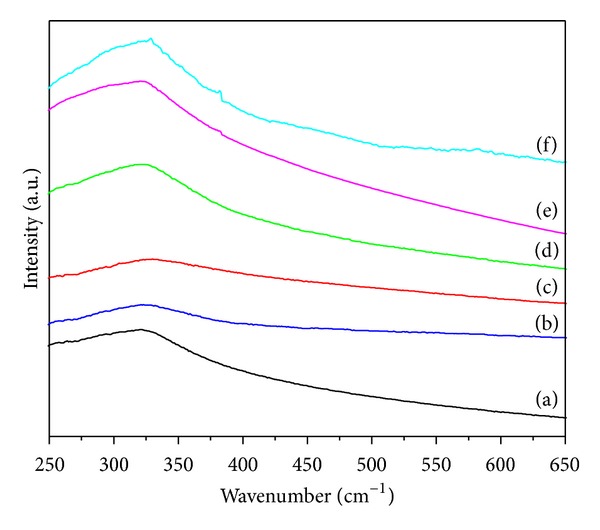
UV-Vis spectra of TiO_2_-NPs synthesized using (a) 0R, (b) 0.25R, (c) 0.5R, (d) 1R, (e) 2R, and (f) 4R.

Figure 6 shows the UV-Vis absorption spectra of the TiO_2_-NPs using different concentrations of rice straw in comparison with the TiO_2_ synthesized using no rice straw as reference. Compared to the very low absorption of the reference TiO_2_-NPs, all the TiO_2_-NPs synthesized using rice straw exhibit a slight shift of the absorption edge toward the visible region. This red-shift originates from decreasing of the particle size of synthesized TiO_2_-NPs.

### 3.6. Surface Properties

**Figure 7:**
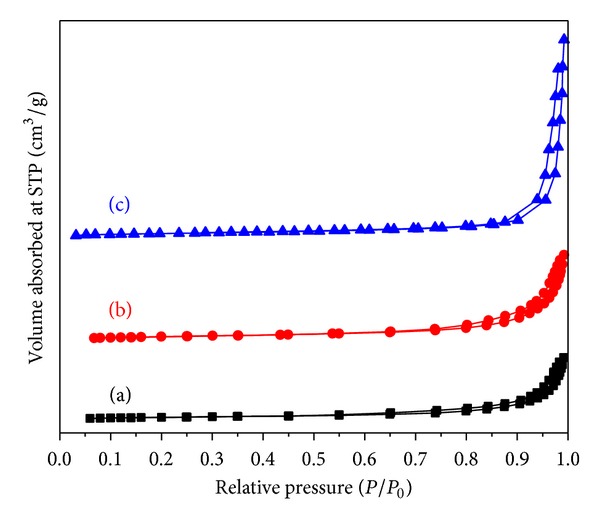
Nitrogen adsorption-desorption isotherms of TiO_2_-NPs synthesized using (a) 0R, (b) 0.25R, and (c) 4R.

The N_2_ adsorption-desorption technique was employed to investigate the effect of rice straw on the surface property of TiO_2_-NPs.  Figure 7 shows isotherms of TiO_2_-NPs synthesized using lowest (0.25R) and highest (4R) amount of rice straw along with reference sample synthesized without rice straw (0R). As seen from Figure 7, all the TiO_2_-NPs exhibited Type IV isotherm which is associated with capillary condensation according to IUPAC classification with Type H3 hysteresis loops. The N_2_ isotherms varied significantly with the rice straw content, indicating that the TiO_2_ porous structure could be controlled by adjusting the amount of rice straw during particle synthesis. Detailed look on isotherms in Figure 6 shows that desorption branch of synthesized samples using rice straw is getting wider compared to sample synthesized without rice straw, representing increase in porosity. In addition, N_2_ adsorption-desorption isotherms have clearly shown gradual enhancement in the volume absorbed through pores of the TiO_2_ samples using rice straw as biotemplate, representing higher pore volumes for template-assisted TiO_2_-NPs. Therefore, it is clear that porosity has been increased during synthesis of TiO_2_-NPs by applying rice straw as biotemplate.

The BET surface area and average pore sizes and pore volumes of TiO_2_-NPs are listed in Table 2. As seen from the table, the average pore size for the as-obtained samples is between 6.5 and 16.5 nm, indicating increase in the pore size of synthesized samples by adding more rice straw. From surface area values, a consequence can be derived that increasing rice straw concentration in synthesis of TiO_2_-NPs could considerably enhance the specific surface area. For example, specific surface area of TiO_2_-NPs synthesized using 4 g rice straw was obtained at 97 m^2^/g, which is more than twofold in comparison with sample synthesized without rice straw. Results from surface properties are in agreement with obtained particle size from TEM since decrease of particle size could lead to surface area enhancement. It is noteworthy to mention that pore structures play an important role as well. As seen from Table 2, pore volumes of TiO_2_ samples improved by increasing the rice straw concentrations, indicating porosity enhancement. In conclusion, surface characteristics of TiO_2_-NPs have been modified using rice straw as biotemplate. Increasing in the surface area and porosity of the synthesized TiO_2_-NPs can result in high photocatalytic properties under UV light irradiation according to report from Budi et al. [32].

## 4. Conclusion

Anatase TiO_2_ nanoparticles were successfully synthesized by conventional sol-gel method based on the template-assisted waste valorization technique using rice straw. Through this method, highly crystalline TiO_2_-NPs with unchanged physical dimensions and minimal agglomeration were prepared. The physicochemical properties of synthesized TiO_2_-NPs were investigated by XRD, TEM, FTIR, Raman, UV-Vis spectroscopy, and surface area and porosity analysis indicating that TiO_2_-NPs crystallize in the anatase phase with smaller size range and high surface area in the presence of rice straw. This soft template is assumed to self-assemble into well-defined aggregated entities which can restrict and direct the growth of the TiO_2_ particles. Modification of the pore volume and size on one hand and decreasing the particle size on the other hand could enhance the surface area of the synthesized TiO_2_ nanoparticles up to 97 m^2^/g which makes TiO_2_-NPs highly potential photocatalyst compared to the titania synthesized using no template. In conclusion, waste valorization approach has dedicated a new pathway to apply sustainable lignocellulosic waste material to fabricate advanced end-products using green chemical technologies.

## Figures and Tables

**Table 1:** The crystalline size of TiO_2_-NPs obtained from Scherer's formula.

Samples	2*θ* (degree)	FWHM (rad)	Size (nm)
0R	25.384	0.0067	20
0.25R	25.385	0.0075	18
0.5R	25.38	0.0090	15
1R	25.346	0.0104	13
2R	25.324	0.0123	11
4R	25.316	0.0135	10

**Table 2:** BET surface area and average pore sizes and pore volumes of TiO_2_-NPs synthesized using different concentrations of rice straw.

Samples	BET surface area (m^2^/g)	Total pores volume (cm^3^/g)	Average pore size (nm)
0R	45	0.09	6.5
0.25R	57	0.12	8.7
0.5R	65	0.13	11.3
1R	73	0.16	14.3
2R	89	0.21	15.0
4R	97	0.23	16.5
